# Predictive factors for responses to primary medical treatment with lanreotide autogel 120 mg in acromegaly: post hoc analyses from the PRIMARYS study

**DOI:** 10.1007/s11102-019-01020-3

**Published:** 2019-12-26

**Authors:** Stephan Petersenn, Aude Houchard, Caroline Sert, Philippe J. Caron, the PRIMARYS Study Group

**Affiliations:** 1 ENDOC Center for Endocrine Tumors, Erik-Blumenfeld-Platz 27a, 22587 Hamburg, Germany; 2 Ipsen, Boulogne-Billancourt, France; 3 Department of Endocrinology and Metabolic Diseases, CHU Larrey, Toulouse, France

**Keywords:** Acromegaly, Predictive factors, Growth hormone, Baseline IGF-1, Hormonal response, Somatostatin receptor ligands

## Abstract

**Purpose:**

PRIMARYS (NCT00690898) was a 48-week, open-label, phase 3b study, evaluating treatment with the somatostatin receptor ligand lanreotide autogel (stable dose: 120 mg/28 days) in treatment-naïve patients with growth hormone (GH)-secreting pituitary macroadenoma. This post hoc analysis aimed to evaluate factors predictive of long-term responses.

**Methods:**

Potential predictive factors evaluated were: sex, age, and body mass index at baseline; and GH, insulin-like growth factor-1 (IGF-1), and tumor volume (TV) at baseline and week 12, using univariate regression analyses. Treatment responses were defined as hormonal control (GH ≤ 2.5 µg/L and age- and sex-normalized IGF-1), tight hormonal control (GH < 1.0 µg/L and normalized IGF-1), or ≥ 20% TV reduction (TVR). Receiver-operating-characteristic (ROC) curves were constructed using predictive factors significant in univariate analyses. Cut-off values for predicting treatment responses at 12 months were derived by maximizing the Youden index (J).

**Results:**

At baseline, older age, female sex, and lower IGF-1 levels were associated with an increased probability of achieving long-term hormonal control. ROC area-under-the curve (AUC) values for hormonal control were high for week-12 GH and IGF-1 levels (0.87 and 0.93, respectively); associated cut-off values were 1.19 μg/L and 110% of the upper limit of normal (ULN), respectively. Results were similar for tight hormonal control (AUC values: 0.92 [GH] and 0.87 [IGF-1]; cut-off values: 1.11 μg/L and 125% ULN, respectively). AUC and J values associated with TVR were low.

**Conclusions:**

The use of predictive factors at baseline and week 12 of treatment could inform clinical expectations of the long-term efficacy of lanreotide autogel.

**Electronic supplementary material:**

The online version of this article (10.1007/s11102-019-01020-3) contains supplementary material, which is available to authorized users.

## Introduction

Acromegaly is a disease characterized by the hypersecretion of growth hormone (GH)/insulin-like growth hormone-1 (IGF-1), typically as a result of a benign pituitary adenoma [1]. Long-acting forms of first-generation somatostatin receptor ligands (SRLs) are a well-established medical treatment for acromegaly. They are recommended in patients who do not achieve an adequate response following surgery, as well as in the first-line treatment of patients who are not suitable for or who refuse surgery [2, 3]. Long-acting SRLs have proven benefits in patients with acromegaly, reducing tumor volume (TV), decreasing GH and IGF-1 levels, and improving comorbidities [4–8]. However, not all patients respond to SRL treatment. In a meta–analysis conducted in 2005, it was found that approximately 50% of patients treated with SRLs achieved hormonal control [9]. One, more recent, systematic review reported considerably lower response rates than this (with an average response rate of 31%) [10], and individual analyses have reported values ranging between 17% and approximately 85% [5, 11]. Potential reasons for these disparities are numerous and include differences in patient populations, lack of standardization of GH and IGF-1 assays, and exclusion of treatment non-responders [5]. These data highlight the importance of being able to predict which patients are likely to respond to treatment with SRLs so as to avoid unnecessary treatment in those unlikely to respond. Previous studies have shown that several factors may influence the response to SRLs. These include: age and sex; pre-treatment and early post-treatment TV, GH, and IGF-1 levels; tumor histopathology (Ki-67, somatostatin receptor subtype 2 expression, AIP expression, granularity, β-arrestin expression); imaging characteristics (T2-weighted magnetic resonance imaging signal intensity); genetic factors; and treatment history [12–30].

PRIMARYS was an international, 48-week, open-label, phase 3b study evaluating primary treatment with the SRL lanreotide autogel (fixed dose of 120 mg/28 days) in patients with GH-secreting macroadenomas [4]. Lanreotide autogel provided early and sustained reductions in TV and GH/IGF-1 hypersecretion. Given its size (90 patients), homogeneous nature (all patients were treatment–naïve), and consistency of dosing (120 mg without dose titration throughout the 48-week study), the study data provide scope for further investigations. Here, we report post-hoc analyses to investigate factors predictive for hormonal control and clinically significant TV reduction (TVR) in patients primarily treated with lanreotide autogel 120 mg/28 days.

## Patients and methods

### Study design, patients, and interventions

The analyses described here were undertaken with the dataset from the PRIMARYS study (ClinicalTrials.gov NCT00690898; EudraCT 2007-000155–34), the methodology of which has been described previously [4].

In brief, PRIMARYS was a 48-week, open-label, single-arm, phase-3b study that was conducted in endocrine centers in nine countries. Men and women (aged 18–75 years) with acromegaly were included in the study if they had a mean GH level (mean of five samples for patients with diabetes mellitus) or nadir GH level (assessed by oral glucose tolerance test, for all other patients) > 1 µg/L, IGF-1 level above age- and sex-matched normal ranges, a macroadenoma (≥ 10 mm diameter), and no visual field defects. Patients were excluded if they had undergone or were likely to require pituitary surgery or radiotherapy, or they had received treatment with SRL, dopamine agonist, or GH receptor antagonist previously, or were likely to require any of these treatments (except lanreotide autogel). Patients were also excluded if prolactin co-secretion was > 100 µg/L.

Patients received a total of 12 doses of lanreotide autogel 120 mg (deep subcutaneous injection every 28 days). Patients could be withdrawn at any time if there was evidence of new visual field abnormalities or other safety concerns, insufficient reduction in IGF-1 levels at week 24 (reduction < 10% compared with the level at baseline, or if in the investigator’s judgement the response was inadequate), or prolactin levels after baseline were > 100 µg/L (for participants with levels of 20–100 µg/L at baseline).

Hormone levels and TVs were assessed centrally at screening, at day 1 (baseline; hormone levels only) and weeks 12, 24, and 48, and at early withdrawal, if applicable; TV at the screening visit was used as the baseline value. The assessments have been described previously [4]. Briefly, TV was measured using pre-specified methods, including magnetic resonance imaging and computer modelling, by three neuroradiologists blinded to the chronology of patients’ scans. IGF-1 levels were assessed at each visit using a radioimmunoassay (Esoterix/LabCorp Endocrine Sciences, CA, USA), and parameters were as follows: lower limit of detection, 7.7 μg/L; lower limit of quantitation, 15 μg/L; intra-assay precision, 5.3–14.1%; and interassay precision, 7.2–17.0%. Five consecutive samples were taken at 10- to 15-minute intervals to assess mean GH levels using a simultaneous one-step immunoenzymatic assay (Access Ultrasensitive GH assay; Beckman Coulter Inc, CA, USA), and parameters were as follows: lower limit of detection, 0.002 μg/L; intra-assay precision, 1.9–3.8%; and interassay precision, 2.7–3.9% [4]. The primary endpoint was the proportion of patients achieving ≥ 20% TVR at last post-baseline value available (LVA). Secondary efficacy endpoints included the proportions of patients achieving GH ≤ 2.5/< 1.0 µg/L, and age and sex-normalized IGF-1 levels at LVA.

### Statistical analyses

Potential predictive factors evaluated were: sex; age and body mass index (BMI) at baseline; and GH, IGF-1, and TV at baseline and week 12. In the present analyses, treatment response was defined as: hormonal control (GH ≤ 2.5 µg/L and normalized IGF-1 levels), tight hormonal control (GH < 1.0 µg and normalized IGF-1 levels), or TV response (≥ 20% TVR) at LVA. Factors predictive for each of these treatment responses were investigated using a series of post hoc analyses. Firstly, potential predictive factors were examined using summary statistics for the proportions of patients achieving hormonal control (both definitions) at LVA according to baseline GH and IGF-1 levels, and the proportions achieving a TV response according to baseline TVs. Secondly, univariate logistic regression analyses were used to examine associations between potential predictive factors (at baseline, week 12, and for changes from baseline to week 12) and each of the three treatment responses. These were followed by correlation analyses with potential predictive factors from the univariate analyses to assess for the presence of multicollinearity among variables. Multicollinearity was detected between baseline and week-12 data, therefore, multivariate logistic regression analyses including baseline and week-12 variables were not performed.

Receiver-operating-characteristic (ROC) curves were then performed. ROC analysis is a well-accepted method that allows predictive accuracy to be tested across the full range of scores and does not require a single, pre-determined cut-off value to determine a true positive result [31, 32]. ROC curves were drawn for each of the three treatment responses, using predictive factors that were significant in the univariate analyses. For each factor in the ROC curves, a cut-off value for predicting a treatment response at LVA was derived by maximizing the Youden index (J = sensitivity + specificity–1). The J measure of sensitivity is a frequently used summary measure of the ROC curve that enables selection of an optimal threshold value (cut-off point) for predictive markers [33]. In the context of the present analyses, this approach minimizes the proportions of false positives (patients with levels below the cut-off value who did not achieve a treatment response) and false negatives (patients with levels above the cut-off who achieved a treatment response).

All analyses were based on data from patients in the intention-to-treat (ITT) population (patients receiving at least one injection of study medication and with at least one baseline efficacy assessment for the primary endpoint [TV] of the PRIMARYS study [4]). All post hoc analyses were hypothesis-generating, and no power calculations were performed. A *p* value of < 0.05 was considered significant.

## Results

A total of 90 patients were enrolled in the PRIMARYS study and received treatment, and 18 of these withdrew due to an insufficient IGF-1 response [4]. At baseline, 47.8% were men, the mean (SD) age was 49.5 (12.4) years, BMI was 27.7 (4.6) kg/m^2^, and time since diagnosis of acromegaly was 121 (150) days [4]. Of the 90 patients, 89 fulfilled the criteria for inclusion in the ITT population, and 88 had LVA data for hormonal response and TV responder status.

**Fig. 1:**
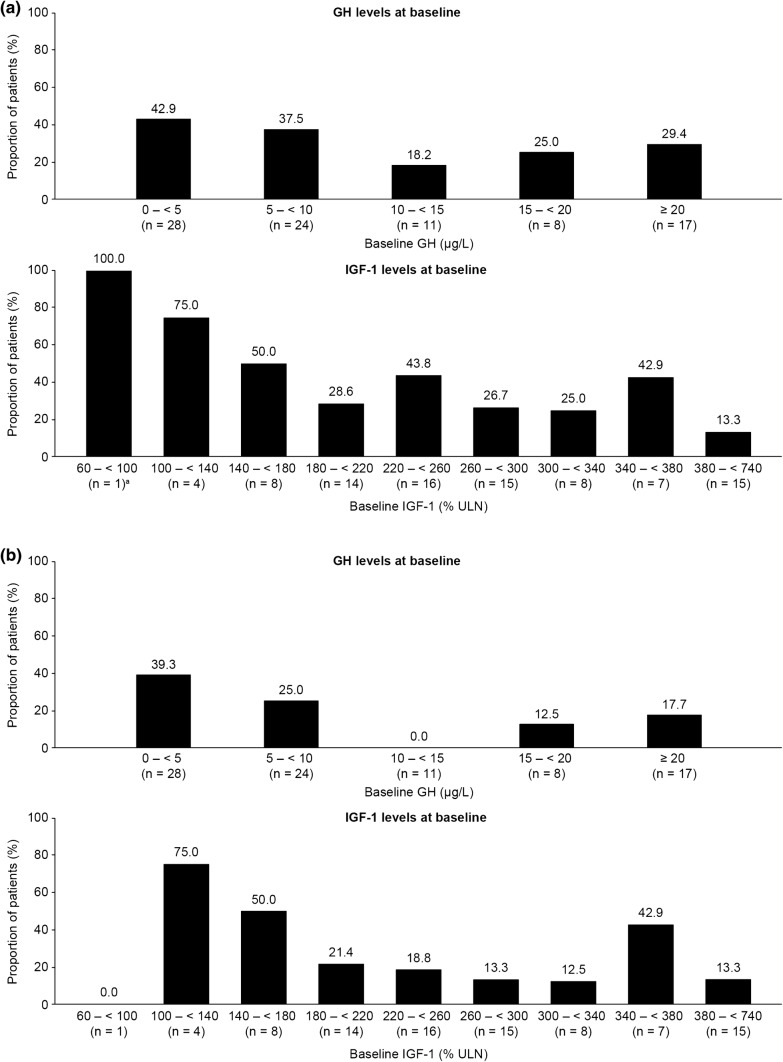
Proportions of patients achieving **a** hormonal control (defined as GH ≤ 2.5 µg/L and IGF-1 levels within normal ranges at LVA) and **b** tight hormonal control (defined as GH < 1.0 µg/L and IGF-1 levels within normal ranges at LVA), at LVA according to baseline GH and IGF-1 levels. *GH* growth hormone, *IGF*-*1* insulin-like growth factor-1, *LVA* last post-baseline value available. Patients with baseline IGF-1 levels between 380 and < 740% ULN were grouped together. Of the two patients who achieved tight hormonal control at LVA, one patient had IGF-1 levels between 460 and 500% ULN, and the other between 540 and 580% ULN. Data are from the intention-to-treat population for patients with LVA data (n = 88)

**Fig. 2:**
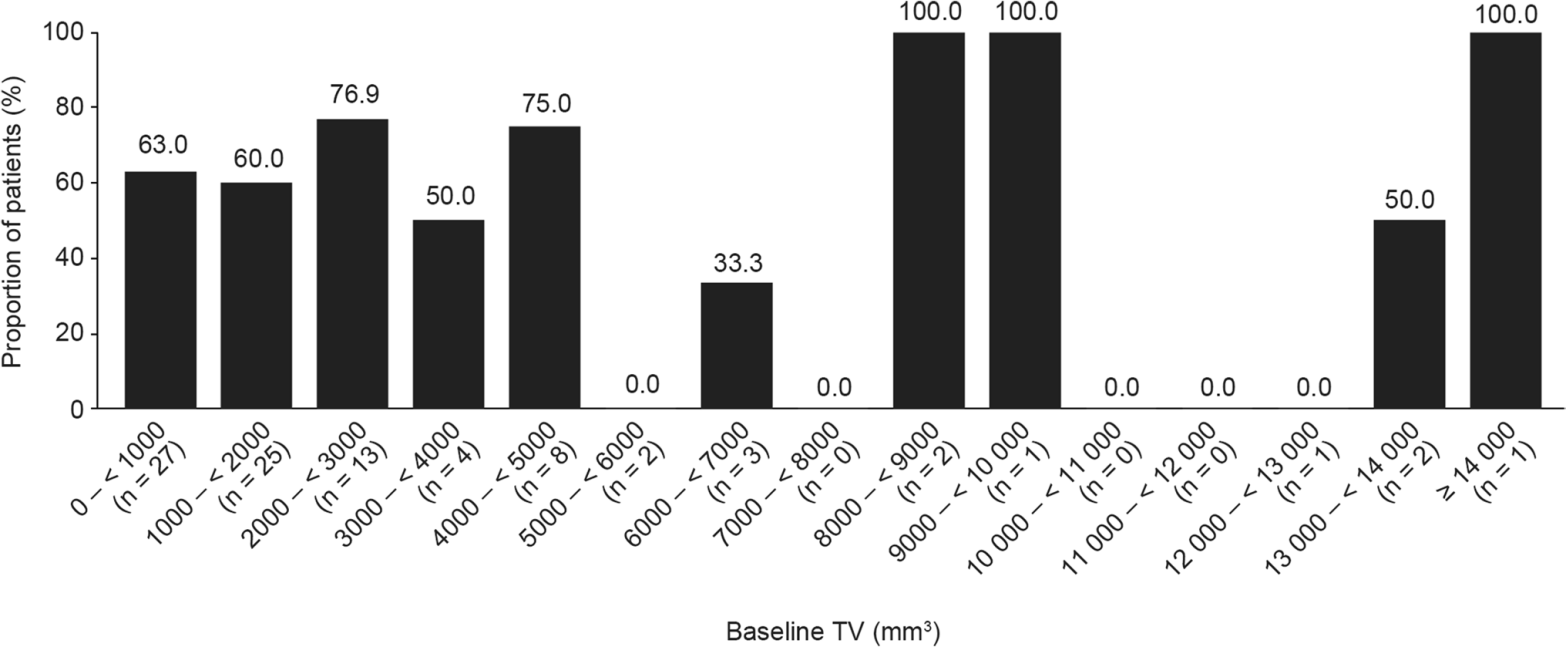
Proportions of patients achieving TV responder status at LVA according to baseline TV. *TV* tumor volume, *LVA* last post-baseline value available. Data are from the intention-to-treat population for patients with LVA data (n = 89). TV responder status was defined as ≤ 20% reduction in TV

As reported previously, the proportion of patients achieving GH ≤ 2.5 µg/L and normalized IGF-1 levels at LVA was 34.1%, while 62.9% achieved a TV response at LVA (a priori analyses) [4]. The proportion achieving tight hormonal control as defined for the present analyses (GH < 1.0 µg/L and normalized IGF-1 levels) was 23.9% (post hoc analysis). Although some patients with higher baseline hormone levels did achieve hormonal control at LVA, the proportions of patients who achieved hormonal control at LVA were generally greater for those with lower baseline hormone levels (Fig. 1a, b). There was no clear relationship between TV responder status at LVA and TV at baseline; however, the majority of patients who achieved a TV response had a baseline TV of < 5000 mm^3^ (Fig. 2).

### Univariate logistic regression analyses

The results of univariate analyses examining the associations between potential predictive factors (baseline, week-12, and change-from-baseline factors) and each treatment response are shown in Tables 1, 2, 3.

**Table 1:** Univariate logistic regression analyses for hormonal control defined as GH ≤ 2.5 µg/L and IGF-1 levels within normal ranges

	Number of patients	Odds ratio [95% CI]	p-Value
Baseline			
** Age (per 10-year higher age)**	**88**	**2.20 [1.39; 3.48]**	**< 0.001**
** Sex (women vs men)**	**88**	**2.87 [1.13; 7.33]**	**0.027**
BMI (≥ 25 vs 20–25 kg/m^2^)	88	0.50 [0.20; 1.26]	0.139
GH (per 1-μg/L lower level)	88	1.01 [0.98; 1.04]	0.433
** IGF-1 (per 50% lower level ULN)**	**88**	**1.28 [1.01; 1.63]**	**0.040**
TV (per 100-mm^3^ smaller size)	88	1.02 [1.00; 1.05]	0.055
Week 12			
** GH (per 1-μg/L lower level)**	**84**	**3.86 [1.87; 7.98]**	**< 0.001**
** IGF-1 (per 50% lower level ULN)**	**85**	**10.70 [3.59; 31.91]**	**< 0.001**
**TV (per 100-mm**^**3**^**smaller size)**	**85**	**1.04 [1.00; 1.08]**	**0.046**
Change-from-baseline to week 12			
** GH (per 10% increase)**	**84**	**0.73 [0.59; 0.90]**	**0.004**
** IGF-1 (per 10% increase)**	**85**	**0.48 [0.33; 0.68]**	**< 0.001**
TV (per 10% reduction)	85	1.37 [0.99; 1.90]	0.059

Factors in bold are statistically significant. Data are based on the number of patients with available data for each factor at each timepoint, and with *p*-values from Chi squared tests

**Table 2:** Univariate logistic regression analyses for tight hormonal control defined as GH < 1.0 µg/L and IGF-1 levels within normal ranges

	Number of patients	Odds ratio [95% CI]	p-Value
Baseline			
** Age (per 10-year higher age)**	**88**	**2.50 [1.46; 4.26]**	**< 0.001**
Sex (women vs men)	88	2.73 [0.95; 7.90]	0.063
BMI (≥ 25 vs 20–25 kg/m^2^)	88	0.74 [0.27; 2.06]	0.566
GH (per 1-μg/L lower level)	88	1.02 [0.98; 1.05]	0.361
IGF-1 (per 50% lower level ULN)	88	1.22 [0.94; 1.58]	0.132
TV (per 100-mm^3^ smaller size)	88	1.02 [0.99; 1.05]	0.125
Week 12			
** GH (per 1-μg/L lower level)**	**84**	**11.61 [3.12; 43.27]**	**< 0.001**
** IGF-1 (per 50% lower level ULN)**	**85**	**4.70 [2.05; 10.81]**	**< 0.001**
TV (per 100-mm^3^ smaller size)	85	1.04 [0.99; 1.08]	0.091
Change-from-baseline to week 12			
** GH (per 10% increase)**	**84**	**0.78 [0.62; 0.98]**	**0.030**
** IGF-1 (per 10% increase)**	**85**	**0.58 [0.42; 0.81]**	**0.001**
TV (per 10% reduction)	85	1.29 [0.91; 1.83]	0.160

Factors in bold are statistically significant. Data are based on the number of patients with available data for each factor at each timepoint, and with *p*-values from Chi squared tests

**Table 3:** Univariate logistic regression analyses for TV responder status

	Number of patients	Odds ratio [95% CI]	p-Value
Baseline			
Age (per 10-year higher age)	89	1.17 [0.82; 1.66]	0.380
Sex (women vs men)	89	1.32 [0.56; 3.12]	0.531
BMI (≥ 25 vs 20–25 kg/m^2^)	89	0.68 [0.26; 1.73]	0.413
GH (per 1-μg/L lower level)	89	0.98 [0.95; 1.01]	0.116
IGF-1 (per 50% ULN lower level)	89	1.02 [0.86; 1.22]	0.794
TV (per 100-mm^3^ smaller size)	89	1.00 [0.99; 1.01]	0.976
Week 12			
** GH (per 1-μg/L lower level)**	**85**	**1.12 [1.01; 1.25]**	**0.039**
** IGF-1 (per 50% lower level ULN)**	**85**	**1.48 [1.13; 1.94]**	**0.004**
TV (per 100-mm^3^ smaller size)	85	1.00 [0.99; 1.02]	0.491
Change-from-baseline to week 12			
** GH (per 10% increase)**	**85**	**0.78 [0.67; 0.90]**	**< 0.001**
** IGF-1 (per 10% increase)**	**85**	**0.73 [0.62; 0.87]**	**< 0.001**
** TV (per 10% reduction)**	**85**	**7.15 [3.15; 16.20]**	**< 0.001**

*BMI* body mass index, *GH* growth hormone, *IGF*-*1* insulin-like growth factor-1, *TV* tumor volume, *ULN* upper limit of normal

Factors in bold are statistically significant. Data are based on the number of patients with available data for each factor at each timepoint, and with *p*-values from Chi squared tests. TV responder status was defined as ≥ 20% reduction in TV from baseline to last post-baseline value available

Associations were significant between three baseline factors and hormonal control, defined as GH ≤ 2.5 µg/L and IGF-1 levels within normal ranges at LVA. Specifically, the odds of achieving hormonal control were 2.20 times higher for each 10-year higher age, 2.87 times higher in women than men, and 1.28 times higher for each 50% lower IGF-1 level ULN (Table 1). No significant associations were identified for BMI, GH levels, or TV. Associations were also significant for 3 week-12 factors: the odds were 3.86 times higher for a 1-μg/L lower GH level; 10.70 times higher for a 50% lower IGF-1 level upper limit of normal (ULN); and 1.04 times higher for a 100-mm^3^ lower TV. Changes from baseline to week 12 in GH and IGF-1 levels, but not TVs, were significantly associated with a treatment response (Table 1).

Only one baseline factor was significantly associated with tight hormonal control, defined as GH < 1.0 µg/L and IGF-1 levels within normal ranges at LVA: the odds of achieving tight hormonal control were 2.50 times higher for each 10-year higher age at baseline. Two week-12 factors were significantly associated with a treatment response for tight hormonal control: the odds of achieving a treatment response were 11.61 times higher for a 1-μg/L lower week-12 GH level; and 4.70 times higher for a 50% lower week-12 IGF-1 level ULN. Changes from baseline to week 12 in GH and IGF-1 levels, but not TVs, were also significantly associated with a treatment response (Table 2).

There were no significant associations between baseline factors and TV responder status at LVA. Associations were significant, however, for 2 week-12 factors: the odds of achieving TV responder status were 1.12 times higher for a 1-μg/L lower GH level and 1.48 times higher for a 50% lower IGF-1 level ULN. No significant association was identified with a 100-mm^3^ lower TV. Changes from baseline to week 12 in GH and IGF-1 levels, and in TVs, were significantly associated with a TV treatment response (Table 3).

### Receiver-operating-characteristic curves

Multiple models were examined for each analysis; however, the simplest model for a given area under the curve (AUC) was chosen according to the ‘parsimony principle’.

#### Hormonal control defined as GH ≤ 2.5 µg/L and IGF-1 levels within normal ranges at LVA

The final model incorporating baseline factors had an AUC of 0.79 (Supplementary Fig. 1). The baseline factors of IGF-1 level, age, and sex were significant in univariate analyses. However, using ROC curves, these factors were associated with relatively poor AUCs: 0.64, 0.74, and 0.63 for IGF-1, age, and sex, respectively. The IGF-1 level cut-off for predicting a treatment response using the Youden index was 225% ULN, but the value of J was low (0.24) and sensitivity and specificity were limited (sensitivity, 0.50; specificity, 0.74). Following multicollinearity between baseline and week-12 factors, and the poor AUCs from baseline ROC curves, the final model selected was based on ROC curves with only week-12 variables.

**Fig. 3:**
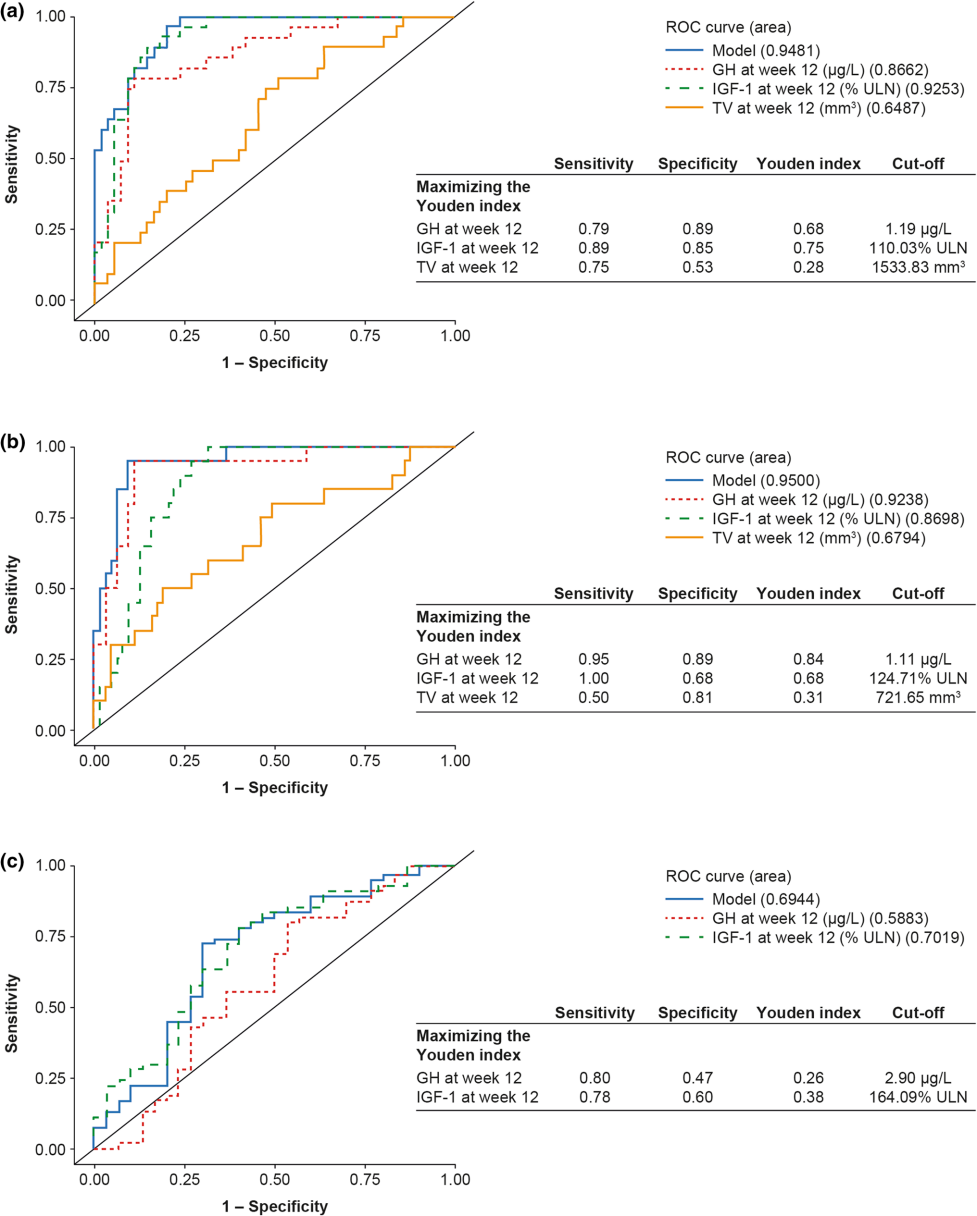
ROC curves drawn for the calculation of week-12 cut-off values for predicting hormonal control and TV responder status at LVA when **a** hormonal control is defined as GH ≤ 2.5 µg/L and IGF-1 levels within normal ranges at LVA, **b** tight hormonal control is defined as GH < 1.0 µg/L and IGF-1 levels within normal ranges at LVA, and **c** TV responder status is defined as ≤ 20% reduction in TV at LVA. *GH* growth hormone, *IGF*-*1* insulin-like growth factor-1, *LVA* last post-baseline value available, *ROC* receiver operating characteristic, *TV* tumor volume, *ULN* upper limit of normal. Data are from the intention-to-treat population for patients with LVA data (n = 88)

The final model incorporating week-12 factors had an AUC of 0.95 (Fig. 3a). Week-12 GH and IGF-1 levels were associated with high AUCs and, with J maximized, were associated with optimal cut-off values of 1.19 μg/L and 110% ULN, respectively (Fig. 3a). In contrast, the AUC for TV in the final model was poor (0.65) (Fig. 3a), and the maximized value of J was low (0.28). This model was not greatly improved by the addition of change-from-baseline factors (AUC of 0.95; Supplementary Fig. 2a) and J values were low. In this model, hormonal control was associated with an optimal cut-off of –55.3% in IGF-1 levels from baseline to week 12 and − 80.3% in GH levels.

#### Tight hormonal control defined as GH < 1.0 µg/L and IGF-1 levels within normal ranges at LVA

ROC curves were not developed for baseline factors as age was the only factor that was significant in univariate analyses.

The final model with week-12 factors had an AUC of 0.95 (Fig. 3b). Twelve-week GH and IGF-1 levels in this model were associated with high AUCs and with optimal cut-off values of 1.11 μg/L and 125% ULN, respectively, with J maximized (Fig. 3b). In contrast, the AUC for TV in the final model was poor (0.68), the value of J when maximized was low (0.31). This model was not greatly improved by the addition of change-from-baseline factors (AUC of 0.95; Supplementary Fig. 2b), and J values were low. In this model, tight hormonal control was associated with an optimal cut-off of − 55.3% in IGF-1 levels from baseline to week 12 and − 72.6% in GH levels.

#### TV responder status at LVA

ROC curves were not developed for baseline factors as none were significant in the univariate analyses.

The final model with week-12 factors had an AUC of 0.69 (Fig. 3c). This model was improved by the addition of change-from-baseline factors (AUC, 0.94); however, the maximized J values were low for percent change-from-baseline to week 12 for both GH and IGF-1 (0.40 and 0.38, respectively, Supplementary Fig. 2C). A TV response was associated with an optimal cut-off of − 68.6% in GH levels from baseline to week 12, − 61.0% in IGF-1 levels, and 21% for TV. Twelve-week GH and IGF-1 levels in the final model (Fig. 3c) were associated with AUCs of 0.59 and 0.70, respectively. Optimal cut-off values to predict TV responder status at LVA were 2.90 µg/L and 164% ULN for 12-week GH and IGF-1, respectively, with J values maximized, but the J values were low in each case.

## Discussion

Post-hoc analyses were undertaken with data from the PRIMARYS study to determine whether treatment responses to lanreotide autogel at 12 months could be predicted from baseline characteristics and/or from week-12 hormone concentrations and TVs. This week-12 information could be useful to clinicians as it could potentially identify “early-response” patients most likely to benefit, thus facilitating individualized management [18, 24], as well as managing the expectations of both patient and physician regarding likely treatment outcomes. The ability to distinguish patients likely and unlikely to respond early in the treatment could also mean that the unnecessary continuation of an ineffective treatment could be avoided in non-responsive patients. Treatment response was based on accepted measures (i.e. hormonal control and TV). Two levels of hormonal control were used (GH ≤ 2.5 μg/L or < 1.0 μg/L, with age- and sex-normalized IGF-1), reflecting the increasing sensitivity and specificity of assays in everyday clinical use [2]. To the best of our knowledge, this is one of the first studies to investigate potential factors at baseline and week 12 after initiation of treatment with lanreotide autogel 120 mg that may predict hormonal control at 12 months.

Older age, female sex, and lower IGF-1 levels were associated with an increased likelihood of achieving hormonal control defined as GH levels ≤ 2.5 µg/L and normalized IGF-1 levels at LVA. This may be related to the pattern of GH secretion in female patients with acromegaly, as well as the age-dependent decline in GH secretion in acromegaly [34]. Older patients were also more likely to achieve tight hormonal control (GH levels < 1.0 µg/L and normalized IGF-1 levels). Pre-treatment IGF-1 levels have also been shown to be an important predictive factor for acromegaly in a study by Bhayana et al., who reported that responders were more likely to have lower baseline levels of IGF-1 [35]. An Italian multicenter retrospective study also demonstrated that pre-treatment IGF-1 levels are predictors for both morbidity and mortality in patients with acromegaly [36].

Twelve-week GH and IGF-1 levels were associated with an increased likelihood of hormonal control, tight hormonal control, and a clinically significant TV response at LVA in univariate analyses. ROC analyses indicated that 12-week GH levels < 1.19 µg/L were predictive for hormonal control at LVA and levels < 1.11 µg/L were predictive for tight hormonal control. Corresponding data for IGF-1 levels were < 110% ULN and < 125% ULN, respectively. At week 12, TV response was not an accurate predictive factor for either hormonal control or tight hormonal control. The results obtained for GH and IGF-1 levels at week 12 resonate with established thresholds for disease control in acromegaly [3]. This model could therefore provide significant value to both clinician and patient by predicting the response to lanreotide autogel 120 mg at 12 months, equivalent to only three injections and with an acceptable safety profile, using data collected in the early stages of treatment (at week 12) [8]. However, patients may still be considered to benefit from lanreotide autogel even if they do not meet these targets for hormonal control if there are nevertheless marked improvements in biomarkers, clinical symptoms, or TV. Maximum effects may occur only after longer treatment periods. Together, the results from this study suggest that early efficacy may be predictive of long-term response; these data reflect the findings of Colao et al. who reported that tumor shrinkage and GH levels after 3 months of treatment with the SRL octreotide long-acting release (LAR), could predict the magnitude of tumor shrinkage at 12 months [37]. A study by Mercado et al. that looked at the efficacy of octreotide LAR treatment at 48 weeks, reported that the majority of patients showed a favorable biochemical response with substantial decreases in GH and IGF-1 levels at week 12. However, the achievement of GH level ≤ 2.5 µg/L and/or the normalization of IGF-1 at 12 weeks of treatment, was not predictive of a significant reduction in tumor volume by the end of study [38]. In 2015, Cuevas-Ramos et al. presented a new structural and functional acromegaly classification [39]. Using cluster analysis, they identified three acromegaly types, based primarily on immunohistochemical and radiological characteristics, with important prognostic implications. Unfortunately, as we do not have follow-up data on surgery and immunohistochemistry for the patients primarily treated with lanreotide autogel in the PRIMARYS study, we are unable to compare results with the proposed classification directly.

The interpretation of data from this study was limited by the post hoc nature of the analyses; the extent to which the results can be generalized to those beyond the study population (previously untreated patients with macroadenomas) is unclear. Furthermore, cut-offs provided here are assay-dependent, which is of special importance for IGF-1, and may be variable. It must also be considered that patients with high baseline GH or IGF-1 levels may have large reductions in hormone levels in response to treatment, but not be considered a responder if they do not reach the cut-off threshold; in this situation, treatment may still be regarded as beneficial. Also, the analysis evaluated only some of the possible predictive factors for response to acromegaly treatment; there are others, such as tumor histopathology (as discussed in the “Introduction”) or expression of somatostatin receptors that may also contribute to how patients respond. A recent post hoc analysis of the PRIMARYS data found that IGF-1 levels after treatment with lanreotide autogel were lower in patients with T2-signal hypointense GH-secreting macroadenomas compared with T2-signal isointense GH-secreting macroadenomas, hence assessment of T2-signal intensity was suggested as a predictive factor for long-term response [30]. It should also be noted that the decision to treat with SRLs may not only depend on predictive factors, but also on the cost of medication and availability of experienced surgeons; both of these factors may vary considerably between countries. Despite these limitations, these analyses are nonetheless valuable as they were conducted with data from a homogeneous population of 90 patients. In addition, both GH and IGF-1 levels and TVs were assessed in a centralized laboratory to reduce measurement variability. The information yielded may assist in the clinical management of treatment and the potential future personalization of therapeutic decisions, likely in combination with other predictive factors [40].

In summary, these post hoc analyses from the PRIMARYS study indicate that treatment-naïve patients with GH/IGF-1 hypersecretion and GH-secreting macroadenomas were more likely to achieve hormonal control at 12 months if they were female, older, or had lower IGF-1 levels at baseline, or if GH levels were less than 1.2 µg/L and IGF-1 levels less than 110% ULN at week 12. Thus, it may in the future be possible to predict as early as week 12 whether treatment-naïve patients with GH-secreting macroadenomas may show a longer-term hormonal response or control to lanreotide autogel 120 mg/28 days.

## Electronic supplementary material

Below is the link to the electronic supplementary material.

Supplementary material 1 (DOCX 784 kb)

## Data Availability

Where patient data can be anonymized, Ipsen will share all individual participant data that underlie the results reported in this article with qualified researchers who provide a valid research question. Study documents, such as the study protocol and clinical study report, are not always available. Proposals should be submitted to DataSharing@Ipsen.com and will be assessed by a scientific review board. Data are available beginning 6 months and ending 5 years after publication; after this time, only raw data may be available.
