# Supplementary material for: Predictive factors for responses to primary medical treatment with lanreotide autogel 120 mg in acromegaly: post hoc analyses from the PRIMARYS study
Source: Pituitary. 2019 Dec 26;23(2):171–81. doi: 10.1007/s11102-019-01020-3 (PMC7066297; doi:10.1007/s11102-019-01020-3)
Supplement: Supplementary file 1 — Supplementary material 1 (DOCX 784 kb) [file 11102_2019_1020_MOESM1_ESM.docx]

**Supplementary material for:**

# **Predictive factors for responses to primary medical treatment with lanreotide autogel 120 mg in acromegaly: *post-hoc* analyses from the PRIMARYS study**

Stephan Petersenn^1^, Aude Houchard^2^, Caroline Sert^2^, Philippe J. Caron^3^, on behalf of the PRIMARYS Study Group

^1^ENDOC Center for Endocrine Tumors, Hamburg, Germany

^2^Ipsen, Boulogne-Billancourt, France

^3^Department of Endocrinology and Metabolic Diseases, CHU Larrey, Toulouse, France

Corresponding author:

Professor Stephan Petersenn, ENDOC Center for Endocrine Tumors, Erik-Blumenfeld-Platz 27a, 22587, Hamburg, Germany

Email: [stephan.petersenn@endoc-med.de](mailto:stephan.petersenn@endoc-med.de)

Tel: + 49 40 401 87985

**Supplementary Fig. 1** ROC curves drawn for the calculation of baseline cut-off values for predicting hormonal control (defined as GH ≤ 2.5 µg/L and IGF-1 levels within normal ranges) at LVA


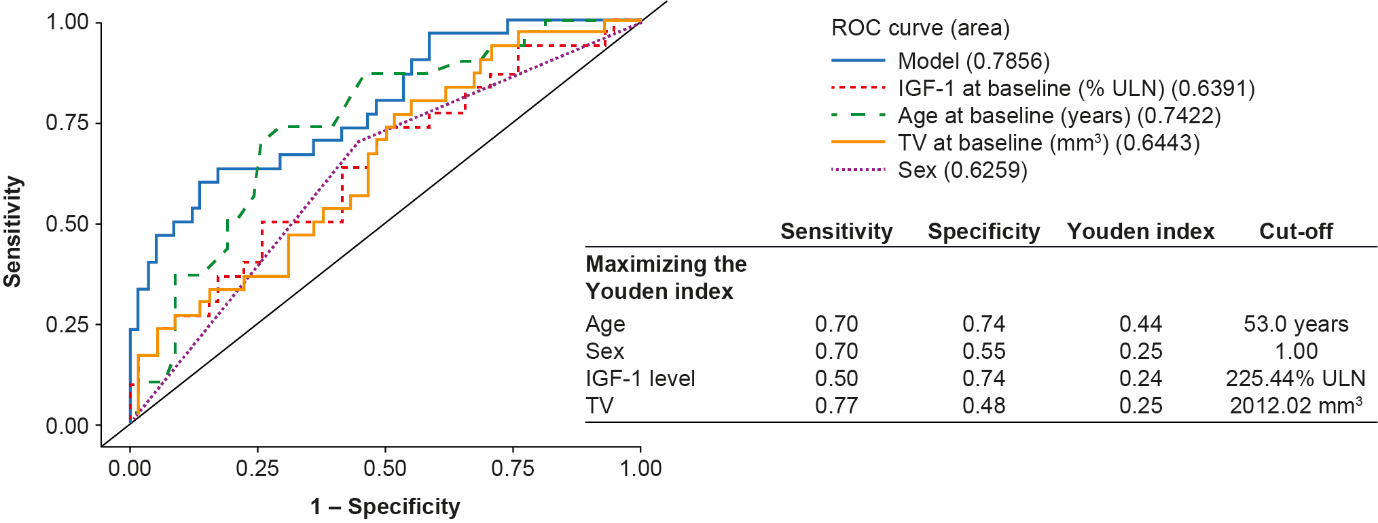


*GH* growth hormone, *IGF-1* insulin-like growth factor-1, *LVA* last post-baseline value available, *ROC* receiver operating characteristic, *TV* tumor volume
Data are from the intention-to-treat population for patients with LVA data (n = 88).
For sex: 1 = male; 2 = female.

**Supplementary Fig.2** ROC curves drawn for the calculation of week-12 and change-from-baseline to week-12 cut-off values for predicting hormonal control and TV responder status at LVA when **a** hormonal control is defined as GH ≤ 2.5 µg/L and IGF-1 levels within normal ranges at LVA, **b** tight hormonal control is defined as GH < 1.0 µg/L and IGF-1 levels within normal ranges at LVA, and **c** TV responder status is defined as ≤ 20% reduction in TV at LVA.


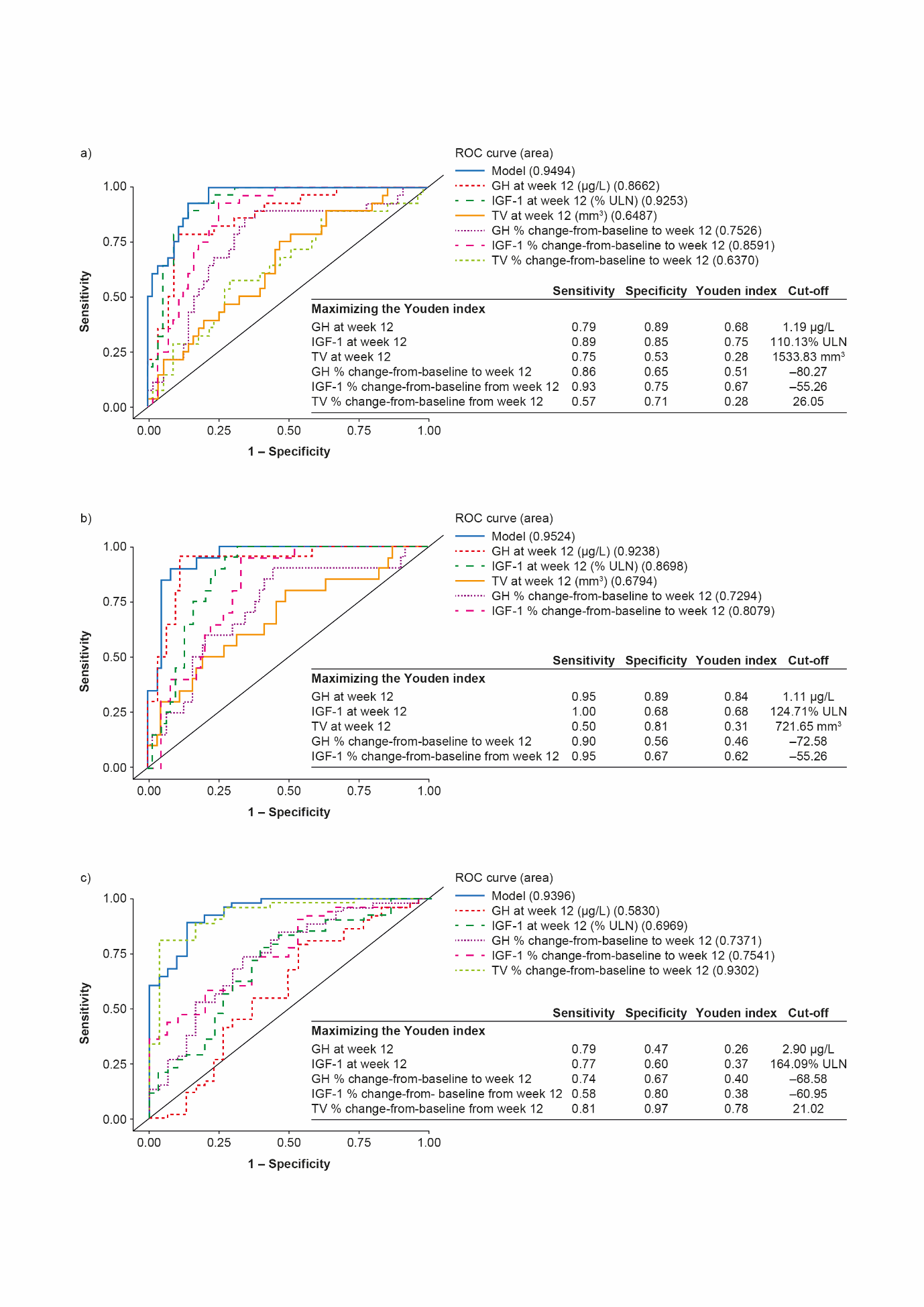


*GH* growth hormone, *IGF-1* insulin-like growth factor-1, *LVA* last post-baseline value available, *ROC* receiver operating characteristic, *TV* tumor volume
